# Pre-conditioning Strategies for Mesenchymal Stromal/Stem Cells in Inflammatory Conditions of Livestock Species

**DOI:** 10.3389/fvets.2022.806069

**Published:** 2022-03-16

**Authors:** Benjamin Uberti, Anita Plaza, Claudio Henríquez

**Affiliations:** ^1^Instituto de Ciencias Clínicas, Facultad de Ciencias Veterinarias, Universidad Austral de Chile, Valdivia, Chile; ^2^Instituto de Patología Animal, Facultad de Ciencias Veterinarias, Universidad Austral de Chile, Valdivia, Chile; ^3^Instituto de Farmacología y Morfofisiología, Facultad de Ciencias Veterinarias, Universidad Austral de Chile, Valdivia, Chile

**Keywords:** mesenchymal stem cells, mesenchymal stromal cells, livestock animals, pre-conditioning, inflammation, hypoxia

## Abstract

Mesenchymal stem/stromal cells (MSCs) therapy has been a cornerstone of regenerative medicine in humans and animals since their identification in 1968. MSCs can interact and modulate the activity of practically all cellular components of the immune response, either through cell-cell contact or paracrine secretion of soluble mediators, which makes them an attractive alternative to conventional therapies for the treatment of chronic inflammatory and immune-mediated diseases. Many of the mechanisms described as necessary for MSCs to modulate the immune/inflammatory response appear to be dependent on the animal species and source. Although there is evidence demonstrating an *in vitro* immunomodulatory effect of MSCs, there are disparate results between the beneficial effect of MSCs in preclinical models and their actual use in clinical diseases. This discordance might be due to cells' limited survival or impaired function in the inflammatory environment after transplantation. This limited efficacy may be due to several factors, including the small amount of MSCs inoculated, MSC administration late in the course of the disease, low MSC survival rates *in vivo*, cryopreservation and thawing effects, and impaired MSC potency/biological activity. Multiple physical and chemical pre-conditioning strategies can enhance the survival rate and potency of MSCs; this paper focuses on hypoxic conditions, with inflammatory cytokines, or with different pattern recognition receptor ligands. These different pre-conditioning strategies can modify MSCs metabolism, gene expression, proliferation, and survivability after transplantation.

## Introduction

Mesenchymal stromal/stem cells (MSCs) have been studied extensively for more than 40 years, with a large body of evidence supporting that MSCs can resolve inflammation and promote tissue repair in various inflammatory conditions, since they can interact with and modulate both innate and adaptive immune responses (1). Many of the mechanisms involved in these processes have been elucidated, but their relevance depending on the cell species and source of the cells remains to be studied, especially in large animals.

The activation of local resident sensor cells of the immune response, responsible for the non-specific detection of microorganisms or tissue damage, leads to the secretion of first-order cytokines that promote the arrival of cells of the adaptive immune response; depending on the inciting cause, local resident sensor cells (epithelial, mast cells, innate lymphoid, and effector memory cells) trigger cellular and humoral effector responses (2). In this sense, the immune and inflammatory response can be modulated at different levels, and MSCs can actively and passively influence both innate and adaptive components of the immune response (3). Recent works in animals deal in-depth with the molecules and pathways that confer MSCs their ability to inhibit lymphocyte proliferation and activation, modify macrophage and lymphocyte polarization, promote cell survival, and therefore modulate inflammation (4–7). However, these mechanisms can be enhanced through different physical and chemical alternatives, which could in turn augment the therapeutic effects of MSCs. The objective of this review is to describe the use of low oxygen tension, pro-inflammatory cytokines or Pattern Recognition Receptors (PRR) as MSCs pre-conditioning mechanisms which could potentially increase their effectiveness and translational potential for inflammatory conditions in livestock animals.

## Improving MSCs Immunomodulatory Function

Low oxygen (O2) tension, high concentrations of inflammatory cytokines, and even presence of microorganisms, are all hallmarks of an inflamed tissue which play a key role in the metabolism of the cells present at the site of injury, and can modulate their functions. It is in these environments where transplanted MSCs will have to perform, by modulating the inflammatory/immune response and promoting the regenerative process. This has led to exploration of ways to pre-condition MSCs in order to “jumpstart” their physiology and strengthen their therapeutic mechanisms, looking to increase the amount of cells that arrive to the site of inflammation and their survival, while also increasing their anti-inflammatory and regenerative effects (8–10). Thus far, pre-conditioning strategies for MSC oriented therapies in veterinary medicine have focused especially on small animals (11–14). In livestock species, studies on MSCs have mostly been published in the last decade (Table 1), with some of their immunomodulatory mechanisms described (Table 2). Much remains to be determined regarding pre-conditioning strategies for MSCs in livestock.

**Table 1:** Effects of mesenchymal stromal/stem cells (MSCs) on inflammatory conditions in livestock animals.

**Animal species**	**Study design**	**Origin**	**Disease**	**Effect**	**References**
Bovine	*in vivo*	AT-MSC	Mastitis	Safety of transplantation. No difference in SCC. Decreased CFU in MSC-treated quarters in an experimental *S. aureus*-induced mastitis model	(15)
	*in vitro* and *in vivo*	AM-MSC	Mastitis	Decrease in *S. aureus* growth and increase in mammary epithelial cell surbival by MSC-CM *in vitro*. No difference between SCC in quarters treated with CM or antibiotics of animals with acute and chronic mastitis	(16)
	*in vitro*	AT-MSC	Fertility	Co-culture of bovine embryos with AT-MSC increases blastocyst development rates and quality when compared to granulosa cells	(17)
Ovine	*in vivo*	BM-MSC	ARDS	Intrapulmonary transplantation of MSC ameliorates airway inflammation induced by LPS, reducing inflammatory cells, proteins, immunoglobulins and inflammatory cytokines in the BALF	(18, 19)
	*in vivo*	UC-MSC	Neuroinflammation	MSCs administered IV reduce cerebral inflammation induced with LPS in preterm fetus, by limiting astrogliosis, improving cerebral cell apoptosis, and myeline content	(20–22)
	*in vivo*	PB-MSC	Wound healing	Locally transplanted MSCs do not increase wound closure but slightly improve neovascularization and skin inflammation	(23)
	*in vivo*	BM-MSC	Tendonitis	Locally transplanted MSCs do not increase wound closure	(24)
	*in vivo*	BM-MSC	Arthritis	MSCs administered IV reduce clinical score and modulate inflammation in a model of arthritis	(25, 26)
	*in vivo*	UC-MSC	Cerebral hypoxia/ischemia	MSCs reduce brain inflammation limiting white matter injury and accelerating self-repair. MSCs also reduce plasmatic levels of TNFα and increase IL-10	(21)
Caprine	*in vivo*	AT-MSC	Mastitis	Small improvement but no significant difference in fibrosis, inflammatory infiltration and cell proliferation before and after MSC transplantation	(27)
Equine	*in vivo*	AT-MSC	Tendonitis	MSCs limit lesion progression, increase organization of collagen fibers, and decrease inflammatory infiltrate. No changes in gene expression	(28)
	*in vivo*	CB-MSC	Wound healing	MSCs transplanted IV do not accelerate wound healing, but do decrease both inflammatory and anti-inflammatory cytokine expression within the wound	(29)
	*in vivo*	CB-MSC	Wound healing	Locally applied MSCs with PRP improve wound healing	(30)
	*ex vivo*	PB-MSC	Skin infection	MSCs reduce viability of methicillin-resistant *S. aureus* biofilms	(31)
	*in vivo*	AT-MSC	Laminitis	Locally transplanted MSCs with platelet-rich plasma improve hoof vascularization	(32)
	*in vivo*	UC-MSC	Osteoarthritis	Intra articular administration of MSCs significantly improved OA clinical scores. No significant improvement from repeated injections	(33)
	*in vivo*	BM-MSC	Osteoarthritis	Improved pain and disability. Additionally, MSC treatment improved cartilage quality	(34, 35)
	*ex vivo*	AT-MSC	Uveitis	Reduction of activation and IFN-γ secretion by T CD 4+ cells isolated from horses with recurrent uveitis. Increase in memory cells	(36)
	*ex vivo*	AT-MSC	Endometritis	Direct injection of MSCs to endometrial slices induce expression of IL-8 and MMP-9	(37)
	*in vivo*	BM-MSC	Endometritis	Reduced uterus inflammation induced by sperm challenge	(38)
Porcine	*in vivo*	BM-MSC	Myocardial infarction	MSCs reduce fibrosis	(39)
	*in vivo*	BM-MSC	Myocardial infarction	MSCs improve cardiac function	(39, 40)
	*in vivo*	BM-MSC	Myocardial infarction	MSCs improve cardiac function	(41)
	*in vivo*	AT-MSC	Renal injury	MSCs and/or their EVs limit renal inflammation, improve oxygenation, fibrosis, renal flow, and glomerular filtration	(42–46)

*AM-MSC, Aminiotic membrane-derived MSC; AT-MSC, adipose tissue-derived MSC; BALF, Bronchio-alveolar lavage fluid; BM-MSC, bone marrow-derived MSC; CB-MSC, Cord blood-derived MSC; CFU, Colony forming unit; CM, Conditioned medium; CT, Cord tissue-derived MSC; EV, extracellular vesicles; OA, osteoarthritis; PB-MSC, Peripheral blood-derived MSC; SCC, Somatic cells count; UC-MSC, umbilical cord-derived-MSC*.

**Table 2:** *In vitro* studies that explore antiinflammatory/immunomodulatory mechanisms and effects of MSCs in livestock.

**Animal species**	**Study design**	**Cell origin**	**Effect**	**Mechanism**	**References**
Bovine	*in vitro*	AT-MSC, BM-MSC	Reduction of S. aureus growth	May be due to the expression of antimicrobian peptides (β-defensin 4A and NK-lysine 1)	(47)
Equine	*in vitro*	AT-MSC, BM-MSC, CB-MSC, CT-MSC	↓ lymphocyte proliferation, ↓ TNF-α, and IFN-γ secretion	BM and CB-MSCs produce NO while AT and CT-MSC does not. Neither of the cell subsets produce Kyneurine, considered as an IDO activity proxy	(48)
	*in vitro*	AT-MSC, BM-MSC, CB-MSC, CT-MSC	↓ lymphocyte proliferation	PGE2 dependent inhibition, while NO inhibition does not restore lymphocyte proliferation	(49)
	*in vitro*	BM-MSC	↓ lymphocyte proliferation and IFN-γ secretion	PGE2 dependent inhibition of proliferation	(50)
	*in vitro*	BM-MSC	↓ lymphocyte proliferation	PGE2 and IDO activity dependent inhibition	(51)
	*in vitro*	BM-MSC	↓ Neutrophil ROS production, no effect on phagocytosis nor NETs liberation	ND	(52)
	*in vitro*	BM-MSC	EVs derived from BM-MSC diminish the effects of pro-inflammatory cytokines on chondrocytes	ND	(53)
	*in vitro*	PB-MSCS	MSCs and MSC CM inhibit the bacterial growth	Antimicrobial petides cystatin C, elafin, lipocalin 2, and cathelicidin	(54)
	*in vitro*	AT-MSC, BM-MSC, EM-MSC	MSCs attenuate *E. coli* growth	Proposed mechanism Lipocalin-2	(55)
Porcine	*in vitro*	BM-MSC	↓ TNF-α by DC, promote M2 macrophage polarization, ↓ lymphocyte proliferation and IFN-γ secretion	ND	(44, 56–58)
	*in vitro*	BM-MSC	MSCs failed to inhibit lymphocyte proliferation, ↑ IL-6	ND	(59)
	*in vitro*	AT-MSC	↓ lymphocyte proliferation	ND	(41)
Lagomorpha	*in vitro*	BM-MSC	↓ lymphocyte proliferation	ND	(60)

*AT-MSC, adipose tissue-derived MSC; BM-MSC, bone marrow-derived MSC; CB-MSC, Cord blood-derived MSC; CM, Conditioned medium; CT, Cord tissue-derived MSC; EV, extracellular vesicles; EM-MSC, Endometrium-derived MSC; IDO, Indoleamine 2,3 Dioxygenase; NO, Nitric oxide; ND, not described*.

### Hypoxia

The issue of optimal culture conditions for MSCs has been under investigation for many years, and the optimum oxygen tension in which to culture cells is an important consideration. Physiological oxygen tension varies greatly between different tissues and can range from 12% in blood to values below 1% (61). MSCs may experience a variety of oxygen tensions, for instance 1–7% in bone marrow or 10–15% in adipose tissue: higher levels of oxygen may lead to early senescence, oxidative stress, DNA damage and lower proliferation (62–64). Regardless of the value, physiological oxygen tension in tissues is significantly lower than the atmospheric tension of the gas generally used in MSCs cultures.

Hypoxic pre-conditioning greatly modifies MSCs physiology, increasing colony formation unit (CFU) number and survival while limiting apoptosis and senescence, mainly but not exclusively through the Hypoxia-Inducible Factor (HIF)-1α mediated pathway (65–68). Some authors have pointed out that these effects could entail an impairment in differentiation capacity (69, 70), but there are mixed opinions on this topic, since other authors describe that low O2 tension culture conditions preserve and even increase MSCs differentiation ability (71–73). Additionally, hypoxia has been shown to increase MSC migration both *in vitro* and *in vivo*, modulating the expression of chemokine receptors and integrins (72, 74–77), and also promoting their vasculogenic potential by increasing expression of VEGF and Angiopoietin (Ang-1) (78–80), both important factors that increase MSCs therapeutic potential. Furthermore, in different pre-clinical models, hypoxic pre-conditioning favors the therapeutic effect of MSCs, enhancing their arrival and engraftment into injured tissue where they promote the survival of tissue resident cells, all of which could be beneficial in clinical settings (76, 81–86). Hypoxic pre-conditioning also enhances MSCs regulation of the inflammatory response by increasing expression of key molecules that modulate the immune response. Hypoxia elevates expression of Indoleamine-pyrrole 2,3-dioxygenase (IDO), HLA-G, Prostaglandin-endoperoxide synthase 2 (PTGS-2), and interleukin (IL)-10 (87–89) and decreases pro-inflammatory molecules such as tumor necrosis factor (TNF)-α, IL-1β and IL-6, and nitric oxide (NO) (87) in different contexts both *in vitro* and *in vivo*, all of which modify cellular fate and condition the inflammatory response by decreasing inflammation and promoting a pro-resolutive context through different pathways (90, 91). Authors have also described that hypoxic pre-conditioning modifies MSCs metabolism and may cause metabolic disruption by increasing glucose uptake and usage, leading to lactate accumulation which attenuates T cell division (92). Interestingly, the MSCs expression of the soluble form of cytotoxic T-lymphocyte-associated protein 4 (CTLA-4) increases under hypoxic conditions, which could affect the lymphocyte activation process by interacting with co-stimulatory molecules of antigen presenting cells (93). Hypoxic priming of MSCs also modifies T cell polarization, by stimulating regulatory T cell proliferation via paracrine mechanisms (89). It is also important to note that hypoxic culture conditions do not impair MSCs ability to respond to inflammatory stimuli nor their immunosuppressive capacities (94). In fact, HIF-1α expression by MSCs induced by hypoxic culture conditions increases their modulatory capacities (95): experimental overexpression of HIF-1α, mimicking hypoxia, has been shown to enhance MSC immunomodulatory properties, while its silencing decreases their immunosuppresive potential (95–98).

There is little information regarding the effect of low O2 tension culture conditions upon MSCs physiology in livestock animals, and mixed results have been reported. In bovines, a recent study shows that culture with low oxygen tension may increase BM-MSCs survival and proliferation with limited effect on gene expression, mainly upregulating the expression of genes related to cell stress, growth, and metabolism (99). Similar results were described in buffalo AT-MSCs, in which hypoxic culture conditions (5% O2) enhanced proliferation, colony formation and differentiation potential, increasing expression of HIF-1α and secretion of basic fibroblast growth factor and vascular endothelial growth factor (VEGF) (100). In sheep, hypoxic culture resulted in faster bone marrow (BM)-MSC population doublings per day, and cell colony formation and viability were not significantly affected (101, 102). Although hypoxia enhanced *in vitro* BM-MSC chondrogenesis (102), this did not translate into increased cartilaginous repair tissue formation following cell transplantation into cartilage defects *in vivo* (103). While some authors report that hypoxic pre-conditioning of porcine MSCs had no effect on proliferation or cell migration (104, 105), others do describe an increase in cell proliferation and impaired osteogenic differentiation in both BM and adipose tissue (AT)-MSCs under hypoxic conditions (106). These conflicting results could be due to differences between culture protocols; for example, Antebi et al. (79) describe that MSCs proliferate significantly faster during 48 h of culture than during 10 days of culture, in both cases under 1% O2 hypoxic conditions. Additionally, authors mention that porcine MSCs cultured under hypoxia had upregulated expression of VEGF and the anti-inflammatory cytokines IL-1 receptor antagonist (RA) and granulocyte-macrophage colony-stimulating factor (GM-CSF), with concomitant downregulation of the pro-inflammatory cytokine IL-8 (79).

In horses, MSCs therapy is of special interest in musculoskeletal diseases such as osteoarthritis, tendon and ligament injuries, bone repair, among others (Table 1). Low oxygen tension culture conditions (5%) attenuate the proliferative capacity of equine AT-MSCs but not BM-MSCs; however, in normoxic (21% O2) conditions a greater proportion of cells were in S phase of cellular cycle, indicating that both cell populations were more active (69). Hypoxic culture seems to keep cells more undifferentiated than normoxic culture, and this is supported by a tendency of hypoxic MSCs to increase expression of embryonic markers (69). This is also described by Griffon et al. (70) who found that hypoxic (5%) culture together with chitosan affected cell yield but improved the stemness of UC-MSCs, with increased expression of embryonic markers such as NANOG, OCT4, and SOX2. In an *in vitro* fracture hematoma model in horses, hypoxic conditions (1% O2) favored survival of MSCs and an increase in osteogenesis, and MSCs survival was correlated with a decrease in live lymphocytes (107).

Hypoxic conditions (1% O2) produce an increase in tenogeneic gene expression in rabbit BM-MSC, which correlates with their increased capacity in promoting patellar tendon repair *in vivo* determined either by both tissue reparation and biomechanical analysis (108). Similar beneficial results are described in a study using hypoxic (1% O2) pre-conditioning in rabbit BM-MSCs used in combination with hyaluronic acid for the treatment of osteoarthritis. Those results show that the addition of hypoxic pre-conditioned BM-MSCs reduce cartilage loss and surface abrasion, with an improvement in histological features compared with hyaluronic acid alone (109). Hypoxic pre-conditioning (1% O2) also enhances the therapeutic effects of rabbit BM-MSC on a disc degeneration model, greatly improving MSC ability to reduce damage and improving extracellular matrix deposition (110).

### Pro-inflammatory Cytokines

MSCs -and even their culture supernatant- can modulate phagocyte functions *in vitro* and *in vivo* without requiring activation (52, 111). However, activation of MSCs with pro-inflammatory cytokines sets in motion several pathways involved in the arrival of MSCs to the injured tissue and is also relevant to development of their full immunomodulatory potential (61). This notion about the need of pro-inflammatory stimuli to activate the immunomodulatory capacity of MSCs is particularly interesting because it could allow identification of the mechanisms required for suppression of the immune response (112). This evidence indicates the potential of MSCs for the treatment of diverse inflammatory conditions in large animals (Table 1).

TNF-α and interferon (IFN)-γ are the most common pro-inflammatory cytokines used for pre-conditioning MSCs, either alone or in combination. IFN-γ is a key modulator of the IDO-Kyneurine pathway which has been shown to be a key component of the immunomodulatory arsenal of MSCs and is involved in several of the reported effects on immune cells (3). In this regard, it is logical to find that pre-conditioning MSCs with IFN-γ results in increased expression of IDO, and of other key immunomodulatory molecules such as prostaglandin (PG)E-2, transforming growth factor (TGF)-β, NO, IL-10, tumor necrosis factor-inducible gene 6 protein (TSG-6) and Programmed death-ligand 1 (PD-L1). These molecules can suppress the cytotoxic activity of natural killer (NK) cells, lymphocyte proliferation and cytokine secretion (113–116), and macrophage polarization (116, 117). Additionally, priming MSCs with IFN-γ has been shown to impair a type 3 immune response characterized by cells that produce IL-17 (118). Regarding function of B cells, pre-treatment with IFN-γ increases MSCs' capacity to reduce B-cell proliferation and immunoglobulin production (119). Some of these effects of IFN-γ are increased with the addition of TNF-α, since the combination of both cytokines increases the expression of immunomodulatory molecules such as IDO, PTGS-2 and inducible nitric oxide synthase (iNOS) (51). This improvement of immunomodulation by MSCs pre-conditioned *in vitro* with IFN-γ and/or TNF-α has also been observed *in vivo* when the cells are transplanted into animals in different preclinical models of inflammatory conditions, such as colitis, diabetes, or graft-vs.-host disease (GVHD) (117, 118, 120–123).

One potential complication of pre-conditioning with IFN-γ is that along with the rise of MSCs immunomodulatory ability there is also an increase in immunogenicity: in horses and rabbits, IFN-γ pre-conditioning has been shown to upregulate MHC class I and II (60, 124), which can be avoided by pretreating cells with TGF-β2 (125). Pre-conditioning with IL-17 could be an alternative, since it not only confers MSCs greater immunomodulatory capacities such as suppression of T cell proliferation and activation, inhibition of type 1 response, and increased induction of Treg cell, but also unlike IFN-γ, it does not induce immunogenicity (126).

In swine, stimulation of MSCs with TNF-α or IL-1 increased their migratory capacity although this effect seems to be dependent on the tissue source (127). Additionally, treatment of porcine AT-MSCs with a IFN-γ, TNF-α and IL-6 cytokine cocktail increased the expression of immunomodulatory molecules such as GBP4, IL1-RA, and IDO while impairing pro-inflammatory cytokines IL-6, IL-17, and TNF-α *in vitro* (128). In horses, MSCs incubation with TNF-α and IFN-γ produces a great increase in the main immunomodulatory paracrine molecules secreted by MSCs such as PTGS-2, IDO, iNOS, and IL-6, while downregulating the expression of other molecules like IL-10, TGF-β1, TSG-6, which are relevant in other species but have no proven role in large animal MSCs immunomodulatory mechanisms (129).

### Pattern Recognition Receptors Ligands

The innate immune system is the first line of defense against microorganisms and is constantly surveying the body for the presence of pathogen-associated molecular patterns (PAMPs), which are detected by highly conserved receptors known as PRR. One of the main PRR families are Toll-like receptors (TLR), and MSCs preferentially express TLR 1-6 (9, 130). Another important PRR family are the nucleotide-binding oligomerization domain (NOD)-like receptor (NLR), whose activation of NLR proteins results in inflammatory responses mediated either by NF-κB, MAPK or Caspase-1 activation, accompanied by subsequent secretion of pro-inflammatory cytokines (131). Ligands for both types of PRR have been considered for pre-conditioning of MSCs, since they not only influence their immunomodulatory functions but also promote antibacterial activity.

The effects on MSCs immune modulation will vary depending on which TLR ligand is used. For instance, Fuenzalida et al. (132) compared the stimulation of TLR-3 and TLR-4 by using polyinosinic-polycytidylic acid [poly(I:C)] and lipopolysaccharide (LPS), respectively. They determined that TLR3 ligands produced a stronger immunosuppressive phenotype compared with MSCs preconditioned with TLR4 ligands, since poly(I:C) induces greater IDO expression which correlates with inhibition of lymphocyte proliferation *in vitro* and improved DSS-induced colitis *in vivo*. Similar results were described by another study in which pre-conditioning with poly(I:C) improved human MSCs immune modulatory properties, decreasing pro-inflammatory cytokines and increasing systemic IL-10 levels in colonic tissues. This was associated with the inhibition of type 1 and 3 immune responses and promotion of Treg differentiation (133).

In horses, there are a few studies about MSCs pre-conditioning using TLR ligands. Cassano et al. (134) showed that TLR-3 or 4 stimulation in MSCs enhances their ability to suppress mitogen stimulated T cells proliferation, with MHC class II positive MSCs having a stronger immunosuppressive activity than MHC class II negative MSCs. Interestingly, MSCs have also been shown to have antimicrobial activity, both by constitutively secreted factors such as defensins, hepcidin and lipocalins, and indirectly by activation of innate immune effector cells (135). Similarly, equine MSCs constitutively express the antimicrobial lipocalin-2 whose expression is augmented by stimulation with LPS, and which could mediate the limitation of the growth of Escherichia coli by MSCs-conditioned media (55). Furthermore, MSCs stimulation with poly(I:C) and [γ-d-Glu-mDAP (IE-DAP)], an NLR agonist, stimulates antimicrobial peptide production and increases bactericidal activity, also suppressing biofilm formation and enhancing neutrophil bactericidal functions (136).

## Conclusion

Pre-conditioning with hypoxia, pro-inflammatory cytokines, or Pattern Recognition Receptors Ligands seem to enhance MSC survival, arrival, engraftment, proliferation, immunomodulatory or pro-regenerative functions, and may increase their therapeutic efficacy. Despite limitations in their use in veterinary medicine, especially due to cost and high variability between species, culture and cryopreservation protocols, and tissue sources, MSCs seem to be a promising alternative therapy for inflammatory and immune-mediated conditions. It is critical to further our understanding of known and novel mechanisms by which MSCs modulate inflammatory processes in livestock animals.

## Author Contributions

BU: manuscript writing and edition. AP and CH: manuscript writing. All authors contributed to the article and approved the submitted version.

## Funding

This study was supported by FONDECYT Grant No. 1210839, Chilean Government.

## Conflict of Interest

The authors declare that the research was conducted in the absence of any commercial or financial relationships that could be construed as a potential conflict of interest.

## Publisher's Note

All claims expressed in this article are solely those of the authors and do not necessarily represent those of their affiliated organizations, or those of the publisher, the editors and the reviewers. Any product that may be evaluated in this article, or claim that may be made by its manufacturer, is not guaranteed or endorsed by the publisher.
